# When Attempts to Help Backfire: Psychosocial Interventions that May Inadvertently Prolong Anxiety Among Youth

**DOI:** 10.1007/s10802-025-01317-x

**Published:** 2025-04-26

**Authors:** Erin E. Dunning, Anika N. Khan, Emily M. Becker-Haimes, Andrew G. Guzick

**Affiliations:** 1 Department of Psychiatry, University of Pennsylvania, Philadelphia, PA USA; 2 University of Pennsylvania Health System, Hall Mercer Community Mental Health, Philadelphia, PA USA

**Keywords:** Anxiety disorder, Children, Adolescents, Treatment, Therapy, Psychotherapy, Accommodation, Harm

## Abstract

Anxiety disorders are among the most common and impairing mental health conditions in children and adolescents. Although exposure-focused cognitive behavioral therapy (CBT) is a well-established treatment for this population based on decades of psychological science, many available psychosocial interventions are not based on this strong empirical foundation. In some cases, interventions for youth with anxiety disorders have the potential to maintain anxiety in the long run. Grounded in a well-developed cognitive-behavioral theoretical frame, this commentary aimed to discuss popular and emerging psychosocial interventions for anxious youth that may inadvertently prolong anxiety. We argue that (1) although the availability of gold-standard CBT (with an adequate focus on exposure therapy) appears to be increasing, it continues to be difficult to access for many youth, (2) several available interventions prescribe avoidance-based strategies that do not enable a child to experience self-efficacy building and corrective learning experiences related to their fears or anxieties, thereby potentially maintaining anxiety in the long-run, and (3) several available interventions are not based in any clear, empirically-supported theoretical frame or evidence base, and thus have unclear benefits for anxiety. In a time when there is increasing alarm about anxiety disorders among youth, building systems that can support tried-and-true interventions based on strong science is of utmost importance. Future research, intervention deployment, and policy efforts should pay more attention to the harms that could arise from psychosocial interventions.

## Introduction

Anxiety disorders are among the most common mental health conditions affecting individuals under the age of 18 years (herein referred to as youth), with recent pooled prevalence rates estimated to be 20.5% (Racine et al., 2021) and lifetime prevalence rates estimated to be 32% by the time a child reaches the age of 18 years (Merikangas et al., 2010). These disorders are associated with high rates of concurrent and future mental health disorders and, without effective treatment, tend to persist into adulthood (Pollard et al., 2023). Furthermore, youth anxiety is associated with impaired outcomes across several domains, including physical health, self-harm, social relationships, education, healthcare, employment, and financial outcomes later in life, as well as high economic costs to society (Langley et al., 2014; Pollard et al., 2023). Thus, effective treatment is essential.

Fortunately, well-established psychosocial interventions for youth anxiety exist. To date, the determination of whether a treatment is considered effective largely has emphasized symptom reduction and improvements in functioning. There is limited empirical work investigating the harm or non-beneficence (herein referred to collectively as harm) within youth anxiety treatment, which represents a critical gap in the current literature (Jonsson et al., 2014). As individuals who are engaged clinically and scientifically in the field of youth anxiety, we aimed to provide a commentary that integrates the current cognitive-behavioral theoretical understanding of youth anxiety and limited extant empirical data to discuss any documented or potentially harmful effects of psychosocial interventions for anxious youth. We considered using a systemic review approach, though given the minimal empirical research on harms in youth anxiety treatments, we believed a commentary that raises potential avenues for further work was more appropriate at this time. For example, one systematic review of harms in psychotherapy trials found that of 132 reviewed studies, only 28 systematically assessed harm, only one of which focused on childhood anxiety disorders (Jonsson et al., 2014). Thus, we consider this review to be a forward-looking and theoretical commentary that we hope will motivate further efforts in this field, rather than a conclusive review of harms caused by treatments for youthwith anxiety disorders.

For our purposes, similar to recent work by McKay and colleagues (2021), we define harm as *the exacerbation or prolonging of anxiety symptoms despite participation in psychosocial interventions for youth anxiety*. Within this definition, we include consideration of both direct harm (i.e., treatment results in worse outcomes than would be anticipated in the absence of treatment, adverse events, etc.) and indirect harm (i.e., loss of resources, prolonged treatment, dampened motivation for future treatment, interference with participation in efficacious treatments, etc.) of psychosocial interventions (Lilienfeld, 2007). Our conceptualization of harm here is guided by cognitive-behavioral theory and our perspectives as clinicians and researchers who conceptualize *maladaptive anxious avoidance* (i.e., the repeated and excessive avoidance of a feared stimuli that is not objectively dangerous) as a hallmark factor responsible for the maintenance of anxiety symptomatology (Baca et al., 2023; Whiteside et al., 2013). Thus, we include treatment effects that result in the perpetuation of maladaptive avoidance as potentially harmful.

We examine harm across three primary domains: harm associated with (1) individual treatments delivered by trained professionals, (2) self-help interventions, and (3) societal trends. The vast majority of identified harms were indirect, and thus the present commentary focuses on the indirect harms with a discussion of a few notable exceptions of direct harms.

## Potential Harms from Individual Therapy Delivered by Professionals

### The Central Role of Exposure Therapy in Cognitive Behavioral Therapy

Cognitive Behavioral Therapy (CBT) is the psychosocial treatment with the strongest empirical support for youth with anxiety disorders, with multiple randomized controlled trials (RCT) demonstrating the superiority of CBT to placebo, waitlist, and psychotherapeutic control conditions (Higa-McMillan et al., 2016). Despite its robust evidence base, within this literature, there is considerable variability in CBT content across trials. Converging literature indicates that the exposure therapy phase of treatment (in which youth are supported to gradually and intentionally face their fears with therapist support) is most key to improving outcomes. Within multicomponent CBT, Guzick and colleagues (2022) found that weekly improvement in Clinical Global Impressions Scale Severity (CGI-S) scores has been found to significantly accelerate with the introduction of exposures (0.11 CGI-S point reduction per session, compared to a 0.066 increase in CGI-S scores during the cognitive skills phase). Similarly, Peris and colleagues (2015) demonstrated that the rate of change in CGI-S score was significantly greater following the onset of exposure exercises (an average monthly change of -1.21 points, 95% faster than the previous treatment period). Further, more time spent on exposure in session significantly predicted Clinical Global Impression Scale Improvement (CGI-I) responder status (OR = 217.01, *p* < 0.001), and encouraging youth to complete more challenging exposure tasks was significantly associated with improved treatment outcomes on the Pediatric Anxiety Rating Scale (PARS) anxiety severity (*b* = -0.870, *p* < 0.001) and the Children’s Global Assessment Scale (*b* = 22.85, *p* < 0.001; Peris et al., 2017). This finding was echoed in a meta-analysis of 75 RCT studies (including 5,412 participants) that found protocols with more exposure produced significantly larger effects (standard mean difference: -0.12 to -0.15; Whiteside et al., 2020). Consistent with this, a recent study demonstrates that “exposure-based CBT” (i.e., CBT that more centrally emphasizes exposures) for youth with anxiety disorders outperforms traditional CBT (which places a similar emphasis on psychoeducation and coping) with regards to reduction in symptom severity at mid-treatment (*p* = 0.001) and post-treatment (*p* = 0.015; Whiteside et al., 2024). Further, “exposure-based CBT” has also been shown to outperform the relaxation-based interventions that are often included as a component of traditional CBT, with higher treatment response rates (57.3% versus 19.2%) and faster and more pronounced symptom reduction (decrease of 0.578 points/week versus decrease of 0.256 points/week) for the exposure condition compared to the relaxation condition (Bilek et al., 2021). 

#### *Indirect Harm of Underemphasizing Exposure in Cognitive Behavioral Therapy*.

It is theoretically possible that indirect harm could result from underemphasizing exposure and overemphasizing coping during CBT. Despite the strong evidence for exposure therapy, therapists often delay, minimize, or neglect exposures entirely (Becker-Haimes et al., 2017; Reid et al., 2018). There are several reasons this pattern may cause inadvertent harm. First, delaying exposure unnecessarily could prolong treatment, further taxing an already overstretched health system and leaving more families on waitlists to start therapy. Second, in many contexts, youth receive far fewer psychotherapy sessions than what is typically delivered in clinical trials, and thus, delaying exposure could mean missing it entirely or not making sufficient progress up the exposure hierarchy. For example, school and primary care-linked therapists can only feasibly provide a limited number of sessions, and even in specialty mental health settings, families often only attend a few visits. By delaying exposure in these cases, youth may miss the critical component of CBT entirely. Third, “watering down” exposure by mixing it with other less potent techniques (e.g., progressive muscle relaxation) has the potential of significantly hampering clinical benefits and minimizing remission from anxiety disorders. For example, the meta-analysis discussed above, conducted by Whiteside and colleagues (2020), found that protocols with more time on relaxation had significantly smaller pre-post effects across independent evaluator, child, and parent informants (Cohen’s *d*: 0.38 to 0.80).

To this end, it is possible that over-emphasizing “coping” could inadvertently lead to the prescription of avoidance or excessive safety-seeking behaviors if not done thoughtfully. Hoffman and Chu (2019) provide a balanced, nuanced discussion of assessing whether a behavior serves a *coping* (i.e., adaptive and supporting approach-oriented goals) or *excessive safety-seeking* (i.e., supporting avoidance, thus perpetuating harm) function. They articulate clear qualities that describe safety-seeking rather than coping function, specifically when the behavior (1) prevents disconfirmation of feared expectations in anxiety-provoking situations, (2) demands excessive attention, (3) leads to misattributions of safety during anxiety-provoking situations, and (4) is used excessively or rigidly (Hoffman & Chu, 2019). Consider a child with academic anxiety who listens to music when feeling anxious about school at home. If they take breaks to listen to music and take some deep breaths when feeling overwhelmed by difficult material before returning to try again in a calmer state, this is likely a *coping* behavior. If, however, the child spends the whole night searching the Internet for new music (used *excessively*), is listening to music loudly while studying (*demanding excessive attention resources)*,or believes they can only tolerate studying if they are distracted by music (*preventing disconfirmation of fears and misattributing success in studying)*, it could also serve a safety-seeking, or avoidance-based, function. If a therapist does not explore this dynamic carefully and instead focuses primarily on coping, it is possible that youth may inadvertently participate in anxiogenic short-term coping. Because the coping phase of CBT is generally the most popular among practicing therapists (Chu et al., 2015; Reid et al., 2018), paying careful attention to the function of these behaviors is critical. That said, anxiety does tend to decline even prior to the exposure phase of CBT (though to a lesser extent; Peris et al., 2015; Guzick et al., 2022), and thus it is unlikely that CBT coping skills would frequently lead to increased avoidance, particularly when delivered by a skilled clinician.

On balance, although there has been an acceleration of research supporting the central role of exposure in CBT, these truly “exposure-based” formats have been far less tested than traditional multicomponent CBT that emphasize a number of skills and techniques, and almost no experimental dismantling studies have been conducted. Thus, more work is needed in this area to definitively show exposure is the active ingredient. More broadly, CBT has been by far the most widely tested therapy for youth with anxiety disorders; it may be that CBT has the strongest evidence base simply because it has been the most studied.

#### Indirect Harm of Adapting CBT to School Settings

Schools are the most common setting for mental health services among youth from the general population, as well as among youth with elevated mental health symptoms and/or clinical diagnoses (Duong et al., 2021). Providing mental health services within schools reduces barriers to receiving care, such as insurance coverage, stigma, and transportation difficulties. Given that schools are uniquely well-positioned to provide equitable access to mental health services to a large proportion of youth, delivering evidence-based practices within the school setting is one promising avenue to increasing the reach of high-quality, effective treatments for youth anxiety. However, if evidence-based practices are not utilized, this has the potential for indirect harm.

The current literature includes several RCTs demonstrating the effectiveness of evidence-based practices, specifically multicomponent exposure-based CBT, delivered within schools in treating youth anxiety (Gee et al., 2020). Recently, there have also been efforts to develop *brief* exposure-based CBT (e.g., reduced number and length of sessions), which may be a better fit for treatment delivery within the school setting. However, at least one randomized noninferiority study to date demonstrates that although *brief* exposure-based CBT was associated with decreases in anxiety symptoms at post-treatment and follow-up, it was not non-inferior to standard exposure-based CBT (Haugland et al., 2020). Although *brief* exposure-based CBT was not shown to be non-inferior to standard exposure-based CBT, given that the protocol demonstrated effectiveness in reducing anxiety, it may be an appropriate alternative in instances where standard exposure-based CBT is not possible. That is, brief exposure-based CBT may reduce the prolonging of anxiety symptoms that may otherwise occur without evidence-based practice.

Despite the evidence demonstrating the effectiveness of exposure-based CBT delivered within school settings, many believe the dissemination and use of these evidence-based practices within schools have been slow and largely constrained to controlled trials (e.g., Mychailyszyn et al., 2011). Unfortunately, it may be that even among school clinicians trained in exposure-based CBT for youth anxiety, sustained use of CBT over time is relatively poor, with one study funding sustained CBT technique use ranging from 41 to 63%, with exposures and parental psychoeducation being the least used components (LoCurto et al., 2020). As discussed earlier, exposures are the key component of CBT, and neglecting exposures entirely can lead to inefficient, ineffective, or, in some cases, even harmful treatment. Thus, these two studies suggest that students are unlikely to receive evidence-based treatment for anxiety at school, and even among those who do participate in CBT, they are unlikely to have the key ingredient included in their therapy experience.

### Exposure Therapy

#### Direct Harm of Exposure Therapy

Although there has been no research we could identify on direct harms of exposure in childhood anxiety treatment, cognitive-behavioral theory, and anecdotal evidence suggests that when applied incorrectly or with limited competence, the exposure therapy component of CBT *could* be directly harmful. For example, prematurely discontinuing exposure tasks in response to a youth’s heightened level of distress prioritizes the youth’s short-term comfort over long-term improvement and perpetuates maladaptive avoidance. Specifically, ending the exposure task early may reinforce anxiogenic cognitions (e.g., “this is very dangerous”), self-efficacy beliefs (e.g., “I cannot handle this), and escape-related behaviors that maintain anxiety, which the exposure task is designed to change. It is worth highlighting that harms have not been reported in studies of exposure to date, likely because clinicians take several steps to minimize these experiences, including through the development of a trusting therapeutic alliance with the therapist, use of incremental exposure along a hierarchy to ensure a child is willing to engage in such tasks, and combining exposure with evidence-based parent coaching and motivational strategies. It has also been argued, however, that harms and adverse events have been inadequately monitored in most psychotherapy research, and thus some harms may be missed in the work that has been conducted to date.

#### Indirect Harm of Exposure Therapy

Some have raised concerns that exposure could harm the therapeutic alliance or lead to drop-out, though to our knowledge these concerns have generally not been substantiated in the literature. Specifically, one study comparing CBT and a support/education-based intervention found that there was no difference in improvement in alliance across the conditions when exposure was introduced in CBT, suggesting that this technique does not rupture the therapeutic relationship (Kendall et al., 2009). Another concern has been that exposure will lead to higher drop-out rates. There have now been two trials we know of comparing exposure-based CBT with traditional CBT (Whiteside et al., 2024) or relaxation (Bilek et al., 2021), neither of which found differences in drop-out, suggesting that exposure is acceptable even when introduced early and centered in treatment. This point is echoed in the childhood obsessive-compulsive disorder literature, in which exposure-based CBT is the norm and results in *lower* dropout than other interventions (Johnco et al., 2020).

### Supportive Therapy

Although less effective than CBT, supportive therapy has been shown to lead to positive outcomes for about half of youth with anxiety disorders (Silk et al., 2018). Supportive therapy is grounded in the common factors of psychotherapy and focuses on empathetic listening and building a strong therapeutic relationship, which are transtheoretical pillars of psychotherapy recognized across mental health disciplines. It is also possible that during supportive therapy, there may be instances in which therapists encourage approach-oriented behavior towards the patient’s goals while empathizing with the difficulty of doing so, similar to what is done in exposure therapy, although not as directly. Accordingly, supportive therapy is a very popular approach for children and adolescents with anxiety disorders (Reid et al., 2018).

#### Indirect Harm of Supportive Therapy

Although, on the whole, supportive therapy does appear to benefit a substantial portion of children with anxiety disorders (Silk et al., 2018), based on the cognitive-behavioral theoretical model, it is theoretically possible that supportive therapy could cause harm in some cases if therapists were to provide unconditional support for anxiogenic cognitions or behaviors. For example, if a child comes to the session and describes how they cannot speak with new people because of how overwhelming it is, and the therapist *only* provides empathy for the intense anxiety they experience in these situations, this may undermine their self-efficacy in gradually approaching social situations and reinforce anxious cognitions (e.g., “I cannot talk to kids in my class because I am an anxious person”). In our clinical experience, many youth advocate for avoiding their anxiety triggers because they believe it will protect their mental health. A supportive therapist who never challenged this notion, and instead focused on providing empathy for their challenges and supporting their coping strategies, may unintentionally participate in prolonging avoidance patterns that have been shown to maintain or worsen anxiety in the long-run. That said, it is important to highlight that one large study of supportive therapy for children with anxiety disorders found that about one half of youth experience meaningful change after a course of supportive therapy (Silk et al., 2018), so although these concerns may ring true in some individual child-therapist relationships, it is unlikely to be the case by and large.

### Play Therapy

Play therapy refers broadly to a class of psychosocial interventions that utilize developmentally appropriate play and games to help children resolve psychological challenges through expressing their thoughts and feelings, exploring the world, and becoming socialized (Koukourikos et al., 2021).

#### Indirect Harm of Play Therapy

Although elements of play are commonly integrated into effective psychosocial treatments for youth anxiety (e.g., gamifying specific cognitive-behavioral exercises like exposures or self-monitoring activities), there is a lack of high-quality empirical evidence (e.g., RCTs) to date demonstrating the effectiveness of play therapy as a monotherapy in addressing youth anxiety, leaving its evidence base inconclusive (see Jensen et al., 2017, for a meta-analysis that failed to identify any RCTs for play therapy published between 2000 and 2010). This is particularly the case among school-age youth as the recent preliminary research, including a few RCTs, focuses on preschoolers (Comer et al., 2019). While one small RCT found preliminary evidence for play therapy reducing symptoms of separation anxiety relative to a waitlist control for preschoolers (*z* = − 3.32, *p* = 0.005; Zarra-Nezhad et al., 2023), another comparing play therapy to family-based CBT found play therapy to result in inferior outcomes with regards to parent-reported sleep problems and co-sleeping behaviors (Kahn et al., 2017). Although it is possible that play therapy could be beneficial for young children, research in this area is quite limited. Because play therapy is relatively unproven, it is possible that favoring it over other evidence-based interventions could also inadvertently prolong anxiety for some youth who could otherwise be participating in gold-standard treatment.

### Mindfulness-Based Interventions

The use of mindfulness-based interventions (MBIs) to treat youth anxiety is increasingly common (Odgers et al., 2020). Although several MBIs have been tested in studies or provided in the community (e.g., mindfulness-based cognitive therapy; mindfulness-based stress reduction), collectively, these therapies share an emphasis on practicing awareness and acceptance of present-moment experiences through experiential exercises (e.g., body scan meditation, Hatha yoga, walking meditation, mindful attention to routine, day-to-day activities).

#### Indirect Harm of Mindfulness

Despite the increasing popularity of mindfulness and MBIs, there is a paucity of empirical support demonstrating how the mechanisms of MBIs address the processes that maintain youth anxiety, as well as limited evidence for the effectiveness of these interventions on youth anxiety. Recent meta-analyses examining the effect of MBIs on youth anxiety symptoms are mixed. For example, a small meta-analysis of 18 articles (including 1,276 participants) found no significant effect of MBIs on child and adolescent anxiety (Cohen’s *d* = 0.013; Ruiz-Íñiguez et al., 2020). However, another small meta-analysis including 14 studies (including 1,489 participants) suggests that they are more effective than passive control conditions (standard mean difference = -0.15, *p* = 0.04), but do not outperform active controls (standard mean difference = -0.13, *p* = 0.09; Zhou et al., 2020). Moreover, a third small meta-analysis of 20 studies (including 1,582 participants) found that MBIs are effective only for select youth (e.g., children [Cohen’s *d* = 0.41], but not adolescents [Cohen’s *d* = 0.21]; Odgers et al., 2020). These meta-analyses that do find a significant effect relative to waitlist controls find only a small overall effect that may have limited clinical significance (Odgers et al., 2020; Zhou et al., 2020). Meta-analytic conclusions are hindered by the inclusion of a majority of non-clinical samples, a relatively small number of included studies, heterogeneity across studies, and a high risk of bias in most studies.

Overall, the extant research fails to demonstrate conclusive support for the effectiveness of MBIs in treating youth anxiety, particularly when compared to other active interventions. Thus, indirect harm may result similarly to those of other ineffective or less effective treatments through their potential to prolong anxiety symptoms despite treatment. In considering MBIs specifically, MBIs also often require considerable time investment on behalf of the child, with a course of treatment involving two to twenty weekly 60 to 90 min sessions and homework expectations ranging from 10 min to one hour per day.

### Creative Arts Therapy

Creative arts therapy is another psychosocial treatment class commonly offered for youth anxiety, which can include dance and movement therapy (DMT), art therapy, or music therapy. DMT is a non-verbal treatment that posits to improve health and well-being through movement (American Dance Therapy Association, 2020). Specifically, given the mind-body connection, movement is believed to “promote emotional, social, cognitive, and physical integration of the individual” (American Dance Therapy Association, 2020). In art therapy, youth utilize art (i.e., painting, sculpting, writing, storytelling, acting, etc.) to communicate with their therapist, express their feelings, and build confidence (Waller, 2006). Music therapists use music interventions to target the physical, emotional, and social needs of youth (American Music Therapy Association, 2010). In treating anxiety, specifically, music is posited to be beneficial because it serves as a distractor that diverts one’s attention from negative to positive stimuli (Lu et al., 2021).

#### Indirect Harms of Creative Arts Therapy

While physical activity has been shown to have beneficial impacts on state anxiety (Carter et al., 2021), to date, there is a paucity of research on the effectiveness of DMT (e.g., for a review, see Parslow et al., 2008), art therapy (e.g., Braito et al., 2022), or music therapies (e.g., Belski et al., 2022) as standalone treatments in reducing symptomatology in youth diagnosed with anxiety disorders. Existing studies are generally hampered by several methodological and statistical factors (e.g., small sample sizes, non-active control conditions), which raise concerns about the replicability of the findings and the generalizability of the results to other anxiety disorders. Thus, potential indirect harms are largely consistent with those described above for therapies with minimal evidence for their effectiveness.

### Sensory Integration Therapy

Sensory Integration Therapy (SIT) was developed within the field of occupational therapy and is typically practiced by pediatric occupational therapy practitioners (Camarata et al., 2020). SIT aims to improve an individual’s sensory integration and regulation through repeated exposure to sensory experiences (e.g., vestibular, proprioceptive, auditory, and tactile inputs), which, in turn, leads to the development of adaptive responses to routine stimuli (Camarata et al., 2020). Anxious youth who present to occupational therapists may be evaluated as having sensory over-responsivity and may receive SIT. Given the high co-occurrence between clinical anxiety and sensory issues (Cervin, 2023), we have often seen SIT prescribed for youth with anxiety disorders in our clinical practice.

#### Indirect Harms of SIT

Although SIT is quite popular among occupational therapists, there has been only preliminary RCT support for its efficacy (Camarata et al., 2020). The few studies that have investigated the efficacy of SIT tend to focus on sensory and functional outcomes rather than anxiety per se, leaving its anxiety-specific benefits inconclusive and theoretically distinct. One study suggested that occupational therapists are more likely to conceptualize behavioral indicators (i.e., from anxiety and sensory scales) and case presentations (e.g., a toddler with Generalized Anxiety Disorder) as representative of a sensory processing disorder (i.e., sensory over-responsivity) rather than an anxiety disorder among toddlers (Ben-Sasson et al., 2007), leading to recommendations of SIT rather than anxiety-specific treatment. Thus, indirect harm is possible due to potential ineffectiveness or increasing risk that a formal anxiety disorder goes undetected. Given how limited research has been in SIT for anxiety disorders, it is unclear whether or not SIT would be a valuable option for youth with anxiety disorders, though it is worth highlighting that SIT was not developed with anxiety in mind as a target specifically.

### Harm Associated with Self-Help Interventions

On the surface, the increasing availability and variety of self-help resources seem favorable, especially given the limited access to professional mental health treatment. Research has demonstrated that self-help treatments offer several benefits, including access to larger and more diverse populations, greater accessibility (i.e., time and place) and convenience, increased autonomy and privacy, greater flexibility, and reduced stigma. These interventions often serve as a useful first step in personalized and stepped-care models of mental health service delivery.

#### Indirect Harm of Self-Help Interventions

The current self-help industry is largely unregulated. This means many available resources lack peer review, are not empirically validated, and can be distributed by unqualified individuals. For example, less than 5% of mobile applications promoted for mental health purposes are estimated to have empirical support (Lecomte et al., 2020). Although legitimate and effective self-help resources exist, the unregulated nature of the self-help industry increases the likelihood that youth and caregivers will unknowingly adopt unhelpful, potentially harmful self-help advice.

##### Bibliotherapy

A brief perusal of bookstores and best-seller lists highlights the recent explosion of the self-improvement industry. Self-help books targeting youth anxiety are widely available to both youth and their caregivers (Rickwood & Bradford, 2012). Given the unregulated nature of the self-help industry, it is not uncommon for self-help books purporting to address youth anxiety to be published by authors without formal mental health training and qualifications and/or to recommend interventions that lack empirical support. Research shows that self-help books rated as having the lowest quality (based on usefulness, scientific support, reasonableness of expectations, amount of guidance in implementing and monitoring treatment progress, and inclusion of harmful advice) were more likely to be written by authors who were not mental health professions, cover multiple problem areas, be based on unorthodox or non-evidence-based treatment approaches, and to make lofty claims not supported by research (Redding et al., 2008). Although we could not identify empirical research on harms from unvetted bibliotherapy tools for youth with anxiety disorders, as described in earlier sections, interventions for youth anxiety that lack empirical support can be ineffective, causing indirect harm by prolonging the length of time that the youth experiences clinically significant anxiety symptoms. However, based on cognitive-behavioral theory, a strong argument can be made that some interventions (i.e., those that promote behavioral or emotional avoidance) may cause harm by exacerbating or worsening symptoms over the long term (although it is worth noting that minimal research has been focused on this issue).

However, there are a number of self-help books written by experts based on their research in this area, several of which have demonstrated some empirical support (i.e., demonstrated efficacy in controlled trials). On the whole, parent-led, although unguided, bibliotherapy following these types of books can provide relief for some families. However, they tend to be less effective than bibliotherapy with support from a therapist or coach. One seminal trial in this area exemplifies this finding well; in an RCT comparing parent-led unguided bibliotherapy, group CBT, and a waitlist condition, bibliotherapy was found to be superior to a waitlist but inferior to group CBT (Rapee et al., 2006). However, when therapists offer brief coaching support in these modalities via occasional phone or telehealth calls, these lower-intensity interventions tend to maintain strong effects relative to in-person CBT (Creswell et al., 2022). Thus, books based in CBT principles should continue to clearly market themselves as a possible first step, rather than an alternative to therapist-led CBT, as a degree of therapist support appears critical to outcomes. If these books are marketed aggressively without thoughtful discussion of this point (as they sometimes are), there is a potential for indirect harm if youth and caregivers believe that the self-help books are equivalent (or even better) than traditional exposure-based CBT, as they may avoid seeking professional treatment (e.g., by perceiving that they “have already learned CBT” or " have tried CBT, and it does not work for me or my child”).

##### Social Media

Social media is another area with a rapid eruption of unregulated self-help advice and resources. While the increased mental health discourse on social media may have some positive benefits, such as increasing social support, it also has the potential to expose youth and caregivers to inaccurate mental health information (Starvaggi et al., 2024). First, social media users and influencers may provide misinformation related to the assessment and diagnosis of mental health disorders. For example, a recent analysis of the most popular TikTok videos (*N* = 100) related to Attention-Deficit/Hyperactivity Disorder (AD/HD) found that 71% of misleading videos misattributed symptoms of anxiety, depression, and mood swings as being specific to AD/HD (Yeung et al., 2022). The dissemination of inaccurate information related to diagnosis has the potential for harm because it can lead youth and caregivers down the wrong assessment and treatment path. For example, if the youth with perfectionism-related school anxiety is exposed to a TikTok video claiming that procrastination and difficulty concentrating during homework are unique symptoms of AD/HD, the youth may conclude that they have AD/HD, rather than understanding that these symptoms can also be indicative of an anxiety disorder.

Second, youth and caregivers will likely encounter inaccurate treatment-related advice on social media. In an analysis of TikTok videos (*N* = 500) tagged with #mentalhealthadvice or #mentalhealthtips, medically trained professionals rated 14.2% of the TikTok videos as including potentially damaging advice (e.g., suggesting medication without consultation of a doctor), 31.4% of videos as containing inaccurate advice, and 83.7% of the videos as “misleading” (PlushCare, 2022). The prevalence of misleading, inaccurate, and potentially harmful treatment-related advice is especially concerning given the strong trust individuals appear to place in these social-media-based recommendations. Social media users and influencers are not only making recommendations for ineffective and potentially harmful interventions, but they are also delivering unsubstantiated warnings against evidence-based interventions. Lorenzo-Luaces et al. (2023) analyzed the top CBT videos on TikTok (*N* = 200) and found that 18.5% expressed negative views of CBT, with the most common critiques being that CBT is ineffective, invalidating, and harmful. Although most videos (73.5%) held positive views of CBT, the negative videos demonstrated more engagement (i.e., comments) and were more likely to be posted by someone who claimed to have undergone CBT (which may have increased their perceived accuracy and authenticity). These types of videos have the potential to harm anxious youth and their caregivers as they may be dissuaded from the gold-standard treatment for youth anxiety.

##### Technology-Based Treatment Programs

The expansion of self-help-related resources, in combination with technological advancements, has led to the development of several asynchronous, technology-based treatment programs for youth anxiety. Multiple meta-analyses have demonstrated that technology-delivered CBT with support from a coach or therapist is more effective than control conditions (Cervin & Lundgren, 2022) and that effects can be comparable to traditional CBT (Donovan & March, 2014).

However, the effectiveness of technology-based treatment programs appears limited to the multicomponent exposure-based CBT programs. For example, in their review, Donovan and March (2014) found that the programs that did not include exposure, focused on only one component of CBT, and/or incorporated depression-focused components were not effective in reducing youth anxiety symptoms. Other computer-based programs have been found to have limited efficacy; for example, a meta-analysis of 36 studies (including 2,692 participants) of cognitive bias modification found a small, non-significant effect (g = 0.16) of this intervention across studies (Sicouri et al., 2024), and bio- and neuro-feedback emotion regulation video games intending to target anxiety have found limited efficacy relative to non-anxiety-focused video game control conditions (Scholten et al., 2016; Schoneveld et al., 2016). Consistent with earlier discussions related to exposure-based CBT versus non-evidence-based treatment, the technology-based treatment programs that neglect exposures may result in ineffective treatment and, thus, prolong the youth’s anxiety disorder. As technology continues to advance and “flashier” technology-based treatment programs continue to be developed (i.e., using virtual and augmented reality or neurofeedback), it is important for youth, caregivers, and practitioners alike not to lose focus on exposure as the critical ingredient to effective treatment for youth anxiety until another is identified.

### Potential for Societal Trends To Maintain or Prolong Anxiety in Youth

#### The Potential for Harm from a Public Health Perspective

Beyond individual treatments, it is also worth noting broader systems, structural, and cultural factors that may ultimately prolong anxiety among youth either by making it disincentivizing to deliver the treatments with the most effectiveness or by emphasizing principles counter to living an approach-oriented lifestyle and reducing opportunities to tolerate uncertainty. As anxiety disorders are among the most common mental health conditions in youth, we likely need to move beyond consideration of one-on-one treatments and consider societal factors that may be exacerbating or contributing to this rise. This next section aims to begin exploring some of the possible structural, cultural, and interpersonal factors in order to encourage more systemic research in this area and inform future public health policies aimed at improving youth anxiety (Frank et al., 2024).

#### Cultural Trends that May Inadvertently Prolong Anxiety among Youth

We highlight four cultural trends that may inadvertently prolong anxiety among youth and acknowledge that each could be a subject of its own separate review: culture of maladaptive avoidance, parenting styles, academic culture and pressure, and social media. We only briefly touch on each here, primarily to discuss them as topics potentially ripe for future systematic research and public health interventions to reduce unwanted harmful effects on youth.

There are broader cultural shifts that could lead to increasing patterns of maladaptive avoidance. A rise in awareness of mental health has led to a greater appreciation for responding to the needs of children who experience significant anxiety. Concurrently, society at large has invested more attention and precautionary efforts towards safety threats to children and families (e.g., bolt locks, alarm systems, school shooter drills, recycling initiatives to combat climate change, etc.). On the whole, these shifts are certainly a net benefit, but it is possible that increased recognition of children’s mental health, as well as safety threats to children, could translate to more entrenched avoidance patterns in some cases. For example, trigger warnings and increased access to virtual learning have made it easier for youth to avoid confronting certain feared situations. Although children entering anxiety-provoking scenarios unprepared and without adequate psychosocial support may react in ways that ultimately sensitize them to these situations (e.g., via escaping from the situation or catastrophizing), perpetuating anxiety in the long run, avoiding them entirely could also perpetuate anxiety.

A recent survey of U.S. parents examining different parenting philosophies found that 45% of parents report being overprotective, while 30% of parents report not pushing their children hard enough, and 35% of parents report giving in too quickly (of note, many parents indicated multiple philosophies; Minkin & Horowitz, 2023), which may reflect changes from the decades prior. While there is substantial cultural variation, philosophies aligned with “permissive” (relaxed parenting) and “helicopter” (overprotective) parenting styles are both generally associated with higher rates of youth anxiety, perhaps because each of these parenting styles tends also to exhibit higher levels of parental accommodation (McLeod et al., 2007).

School is another domain, albeit less well studied, where accommodation of youth anxiety occurs. There are laws, such as the Individuals with Disabilities Education Act and Section 504 of the Americans with Disabilities Act, that ensure that students whose anxiety significantly impacts their academic or social functioning at school are provided with support, accommodations, and modifications. These school-based supports are crucial to ensuring that the student can participate and be successful within the school setting. However, when school-based supports facilitate long-term avoidance of anxiety-provoking stimuli in the absence of concurrent approach-oriented anxiety management, they may inadvertently maintain the student’s anxiety. Several studies have highlighted that avoidance-based strategies for anxiety are very common in these accommodations, without a concurrent focus on incremental approach-oriented coping (Green et al., 2017; Conroy et al., 2022; Phillips et al., 2022).

In recent years, there also has been an increasing push for youth to attain higher levels of education. The number of high schoolers who immediately enroll in college has increased substantially in the past two decades (Schaeffer, 2022; Table 302.60, NCES, 2023), and high school students who are engaging in preparatory high schools tend to exhibit higher levels of psychological stress as well as symptoms of anxiety and depression than those who or not (Feld & Shusterman, 2015). Furthermore, greater anxious distress in youth within higher education is associated with greater perceived academic pressure (Wuthrich et al., 2020), which may be best conceptualized as resulting from systemic issues. These cultural shifts and societal messages about the critical importance of a college education may be worth conceptualizing as a potential public health harm as it relates to the exacerbation of youth anxiety.

Finally, we would be remiss if we did not acknowledge the potential impact of smartphone use and social media on increasing youth anxiety. Despite widespread panic about the potential contribution of social media and smartphones to a “mental health epidemic” in young people, which often centers on anxiety, research on this connection is surprisingly mixed, with many studies finding a minimal association (see Odgers, 2024, for a discussion). This field has been highly limited by minimal experimental or longitudinal studies; thus, while there is almost certainly an association between smartphone or social media use and certain domains of mental health for a meaningful proportion of youth, conclusive evidence is still lacking. Public policy as well as systematic research on smartphone use and youth anxiety are areas of important future consideration.

#### Potential Challenges Stemming from the Structure of the Mental Health System

It is also worth highlighting the structural factors that may limit the reach of evidence-based treatments and perpetuate delivery of ineffective or harmful treatments. First, providers who operate within the public mental health system are subject to low reimbursement rates and other financial stressors (Stewart et al., 2016). Such providers are primarily those with a master’s degree (HRSA, 2023) who may have received limited to no formal training in anxiety diagnostics or effective treatment practices (Becker-Haimes et al., 2019). These clinicians are often pushed to high productivity standards that limit time for thoughtful session planning (Last et al., 2024), which are often necessary for leading anxiety treatments. Indeed, higher caseloads are associated with reduced use of effective treatment for youth anxiety (Becker-Haimes et al., 2017). The administratively cumbersome and costly nature of paneling with insurance companies likely actively disincentives many anxiety specialist therapists from taking any insurance plans at all (Frank et al., 2024; Becker-Haimes et al., 2020). This drives patients with fewer resources to pay out of pocket to receive care from providers working in the public sector who are more likely to use ineffective or harmful treatment practices.

Second, the fractured nature of our mental health system has led to little systematic oversight into what treatments are delivered. Incentive structures have not been developed for practitioners to use evidence-based therapies, or to demonstrate positive change among their clients. Building in benefits to therapists who demonstrate the efficacy (and lack of harm) for their clients could be one avenue to minimize harms from psychosocial treatments.

## Discussion

Several themes emerged across our commentary of how psychosocial interventions for youth anxiety have the potential for unintended consequences. First, even within CBT, the gold-standard treatment for youth anxiety, we believe the potential for harm exists. Our first primary source of harm is the inconsistency within the delivery of the intervention. There is considerable variability in the emphasis/inclusion of various components, with the most effective component (i.e., exposures) often underutilized and the least effective component (i.e., relaxation) frequently overutilized (Becker-Haimes et al., 2017; Reid et al., 2018). Youth engagement in CBT that results in either no change or a worsening of symptoms may be particularly harmful, as youth and families may lose hope after concluding that even the gold-standard treatment does not improve anxiety symptoms. The second primary source of harm is the limited availability of the most evidence-based treatment. Due to the relatively low number of CBT-trained clinicians who practice primarily in out-of-pocket, specialist settings, access to these services is quite limited (Reardon et al., 2020). Even among those with access to CBT, the days, weeks, months, and even years spent on waitlists prolong youth anxiety symptoms and add considerable time to illness duration. Further, in the United States, these services are often unattainable as the majority of child anxiety specialists bill out-of-pocket due to the existing reimbursement infrastructure. Relatedly, we believe that the lack of access to CBT is, in part, to blame for the large proportion of anxious youth who end up participating in the many non-evidence-based treatments reviewed in this paper that have the potential to prolong anxiety. Both the inconsistency of CBT delivery and lack of access are identified problems currently being examined by researchers, advocates, mental health professionals, and policy-makers. Future research should continue to increase the efficiency and consistency of dissemination and implementation of exposure-based CBT. Successfully resolving these barriers will likely require a multi-pronged approach with collaboration between researchers, clinicians, policymakers, legislatures, and insurance companies.

Second, we believe that many available interventions delivered in healthcare, school, and family contexts adopt an avoidance-based orientation for managing youth anxiety. As a result, several self-care/coping strategies (e.g., journaling, meditating, listening to music), family responses, and school-based supports risk promoting emotional and/or behavioral avoidance rather than approach-oriented coping. As discussed throughout the article, avoidance is conceptualized by cognitive-behavioral theory as the primary factor that maintains anxiety; thus, promoting avoidance results in the maintenance and, often, worsening of youth anxiety. Future research is warranted to develop and test methods for disseminating information about the approach orientation for anxiety management and embedding approach-oriented coping across psychosocial interventions. For example, potential methods include partnering with and training key stakeholders, developing social media campaigns, and incorporating relevant training into continuing education seminars for clinicians and teachers.

Third, the wide variety of intervention options available to youth and families may not be beneficial. Throughout our review, we discussed multiple face-to-face therapy options, technology-based therapy programs, and self-help books, which ranged in their levels of effectiveness, research support, theoretical cogency, and potential for harm. Unfortunately, given caregivers’ lack of help-seeking knowledge (e.g., Reardon et al., 2018; Reardon et al., 2020), the multitude of available choices is likely to be overwhelming—*Which treatment do I choose? Which book should I read? Should I try this anxiety video game?* Further, caregivers have the potential to unknowingly choose one of the available but ineffective options. Researchers and clinicians considering developing a novel treatment protocol, designing a new technology-based therapy program, or adding to the ever-growing self-help literature should thoughtfully consider whether the value added by this additional choice outweighs the increased decisional burden it places on caregivers. Further, policymakers are tasked with changing incentive structures to optimize the chances that evidence-based options are available to all families.

Fourth, our understanding is that there is a lack of empirical data on the harmful effects of psychosocial interventions. While much of the research has focused on demonstrating the efficacy of therapeutic interventions compared to placebo, waitlist, and psychotherapeutic control conditions, few have tested the harmful impacts of psychosocial interventions on youth anxiety. This is a fruitful area of potential research, as there is much to be learned. The arguments made throughout this paper for potential harm in these interventions were primarily theoretical and indirect (e.g., delaying access to treatment or prolonging anxiety). Potential questions include: How does the length and severity of illness differ among youth who receive effective versus ineffective treatment? What are the differences in current and prospective outcomes between youth who received CBT with exposures versus those without exposures? How does school performance compare among youth who received approach-oriented versus avoidance-oriented school-based supports? In addition to opportunities for increased beneficence and non-maleficence that the knowledge from these studies could offer, the existence of an empirical body of literature investigating harm in youth anxiety treatments would allow future researchers the ability to aggregate the information in a systematic review and/or meta-analysis.

It is important to acknowledge that several limitations exist within the present commentary. First, we did not utilize a systematic review methodology (e.g., systematic review and/or meta-analysis) of the literature given how rarely empirical research investigates harm within youth anxiety treatment. Because of this, any arguments or implications we drew from this commentary are based on our intrinsically biased knowledge of the evidence base and theoretical lens. It is possible that there are many relevant studies we neglected that could undermine our arguments. We hope that the points raised in this paper will stimulate more systematic research and advocacy that ultimately seeks to minimize harms for youth with anxiety disorders, but it should not be used to make definitive or conclusive arguments about harms in childhood anxiety disorder treatment.

Second, we chose to adopt a relatively narrow scope regarding our specific definition of harm, which is consistent with the medical model (i.e., the prolonging or exacerbation of anxiety symptoms). Regarding our chosen definition of harm, it is critical to acknowledge that other important forms of harm exist that warrant future consideration. First, psychosocial interventions may cause unique forms of harm to anxious BIPOC youth (e.g., cultural oppression; Sue, 2015). This may be especially relevant given the structural racism that afflicts healthcare, the deeply rooted implicit biases, and the fact that most research samples are comprised of white, Euro-American youth. Although the few studies that have investigated this question have not consistently found an effect of race or ethnicity on treatment outcomes, BIPOC youth are highly underrepresented in most of these samples, and thus there is unlikely to be sufficient statistical power to detect potential effects (Norris & Kendall, 2021). Second, psychosocial interventions may cause harm to minoritized populations (outside of the patient) if the interventions reinforce stereotypes and negative stigma (e.g., Pinciotti et al., 2022). Third, psychosocial interventions may have harmful public health implications, such as extended time away from school (or work for caregivers), lost productivity, and high costs associated with care.

Third, given that we chose not to complete a systematic review due to the limited extant literature investigating harm within youth anxiety treatment, we chose to restrict youth anxiety treatments to select psychosocial interventions that are most common and salient in our research and clinical practice. Thus, this approach introduces bias, given that it is based on our own experiences and opinions. Additionally, several proposed psychosocial interventions (e.g., equine-assisted therapy) that warrant future review were excluded from this commentary. Further, while our focus was on psychosocial interventions, subsequent future work should also review harm within non-psychosocial treatments for youth anxiety, including psychopharmacological interventions, medical marijuana, nutritional and supplemental treatments, and non-invasive brain stimulation techniques (e.g., transcranial magnetic stimulation).

## Conclusion

There is a growing public panic about an “anxiety crisis” in children and adolescents. Increased awareness of anxiety among youth is a wonderful development in our culture. Many efforts directed toward this problem have the potential to help children and adolescents manage or even overcome clinically significant anxiety. However, in this paper, we argue that many such efforts could be ineffective, inefficient, or even harmful. Our field has developed a robust psychological understanding of anxiety in childhood, yet many popular and emerging psychosocial accommodations and interventions we discussed in this paper are not based on this evidence base. In many cases, these interventions emphasize avoidance without paying attention to incremental exposure to feared or anxiety-provoking situations, which could compound problems in many cases. It is critical to build systems that facilitate tried-and-true interventions for youth struggling with anxiety or, when not feasible, are offering accommodations based on scientifically supported, common sense principles. Future research, intervention deployment, and policy efforts should pay more attention to the harms that could arise from psychosocial interventions.

## Data Availability

Not applicable.
